# A population of wheat multiple synthetic derivatives: an effective platform to explore, harness and utilize genetic diversity of *Aegilops tauschii* for wheat improvement

**DOI:** 10.1007/s00122-018-3102-x

**Published:** 2018-04-28

**Authors:** Yasir Serag Alnor Gorafi, June-Sik Kim, Awad Ahmed Elawad Elbashir, Hisashi Tsujimoto

**Affiliations:** 1 Arid Land Research Center, Tottori University, 1390 Hamasaka, Tottori, 680-0001 Japan; 2 Agricultural Research Corporation, P. O. Box: 126, Wad Medani, Sudan; 3 RIKEN Center for Sustainable Resource Science, Tsukuba, Ibaraki 305-0074 Japan

## Abstract

**Key message:**

The multiple synthetic derivatives platform described in this study will provide an opportunity for effective utilization of *Aegilops tauschii* traits and genes for wheat breeding.

**Abstract:**

Introducing genes from wild relatives is the best option to increase genetic diversity and discover new alleles necessary for wheat improvement. A population harboring genomic fragments from the diploid wheat progenitor *Aegilops tauschii* Coss. in the background of bread wheat (*Triticum aestivum* L.) was developed by crossing and backcrossing 43 synthetic wheat lines with the common wheat cultivar Norin 61. We named this population multiple synthetic derivatives (MSD). To validate the suitability of this population for wheat breeding and genetic studies, we randomly selected 400 MSD lines and genotyped them by using Diversity Array Technology sequencing markers. We scored black glume as a qualitative trait and heading time in two environments in Sudan as a quantitative trait. Our results showed high genetic diversity and less recombination which is expected from the nature of the population. Genome-wide association (GWA) analysis showed one QTL at the short arm of chromosome 1D different from those alleles reported previously indicating that black glume in the MSD population is controlled by new allele at the same locus. For heading time, from the two environments, GWA analysis revealed three QTLs on the short arms of chromosomes 2A, 2B and 2D and two on the long arms of chromosomes 5A and 5D. Using the MSD population, which represents the diversity of 43 *Ae. tauschii* accessions representing most of its natural habitat, QTLs or genes and desired phenotypes (such as drought, heat and salinity tolerance) could be identified and selected for utilization in wheat breeding.

**Electronic supplementary material:**

The online version of this article (10.1007/s00122-018-3102-x) contains supplementary material, which is available to authorized users.

## Introduction

Bread wheat (*T. aestivum* L.) originated through a few events of natural hybridization between durum wheat (*Triticum turgidum* L. subsp. *durum*) and *Ae. tauschii* Coss. Because these events involved few progenitors, the genetic diversity of durum wheat and *Ae. tauschii* is not fully represented in the current bread wheat germplasm (Dreisigacker et al. 2008; Li et al. 2014). This narrow genetic diversity limits that availability of QTLs and genes useful for wheat breeding to face the projected demand for food (Tilamn et al. 2011). One approach to introducing new genetic diversity of wheat progenitors into the cultivated bread wheat gene pool is to develop and use primary synthetic (PS) hexaploid wheat (Mujeeb-Kazi et al. 1996). PS lines are amphiploids resulting from interspecific crosses between diploid *Ae. tauschii*, donor of the D genome, and modern durum or emmer wheat (*T. turgidum* L. subsp. *dicoccum*), donor of the A and B genomes (Jafarzadeh et al. 2016). Since the 1980s, about 1200 winter- and spring-habit PS lines have been developed at the International Maize and Wheat Improvement Center (CIMMYT) (Van Ginkel and Ogbonnaya 2007) and used to capture considerable genetic diversity from progenitor genomes (Mujeeb-Kazi et al. 1996; Zhang et al. 2005). The practical value of this diversity can be seen in the resistance to a range of biotic and abiotic stresses that has been used in wheat breeding (Lopes and Reynolds 2011; Ogbonnaya et al. 2013; Jafarzadeh et al. 2016).

The current approach to use or explore the genetic diversity of wheat progenitors through identification and evaluation of promising PS lines and then to cross the selected PS lines with elite wheat cultivars is not efficient and fast enough. In this approach only a limited number of PS lines can be used, it is difficult to evaluate yield potential traits at the PS level, and such estimates are not reliable. Moreover, Ogbonnaya et al. (2013) reported that the expected traits of PS may not always appear in backcross progenies with the elite wheat cultivars due to the large genetic differences between the genetic backgrounds of the PS and the elite wheat cultivars, and therefore concluded that *Ae. tauschii* genes should be evaluated in the genetic background of elite wheat cultivars using populations including large diversity of *Ae. tauschii* in background of elite cultivars. Thus, a new approach or platform for efficient exploration, harnessing and utilization of this tremendous genetic diversity is needed.

Recent advances in genomics, easy access to abundant information on genetic markers and availability of wheat genome sequences have paved the way for identification of genetic factors underlying complex phenotypes. This knowledge will facilitate efficient development of new cultivars through marker-assisted breeding and genomic selection. Taking advantage of molecular marker availability, a new strategy was developed for efficient gene mining and QTL identification in wheat wild relatives, which is expected to contribute to wheat germplasm enhancement. Using this strategy, a population harboring genomic fragments from the diploid wheat progenitor *Ae. tauschii* in the background of bread wheat was developed by crossing and backcrossing 43 synthetic wheat lines with the common wheat cultivar Norin 61. We named this population multiple synthetic derivatives (MSD). Previously, we grew 1000 plants from the MSD population under heat stress conditions in Sudan and selected six lines visually as potential heat-tolerant lines. We evaluated these lines in the field and under growth-chamber conditions and confirmed two lines as heat tolerant (Elbashir et al. 2017a). We also evaluated the 400 lines used in this study in four different heat stress environments in Sudan and selected several highly heat-tolerant lines and several other lines that contained alleles able to enhance yield potential (Elbashir et al. 2017b). This paper describes the MSD population strategy, reports the validation of this population and its usefulness for wheat breeding, QTL analysis and mining for genes originating from the wheat progenitor *Ae. tauschii*.

## Materials and methods

### Production of the MSD population

To produce the MSD population, we used 44 PS wheat lines as donors and the bread wheat (*T. aestivum* L.) cultivar ‘Norin 61’ (N61) as a recipient. The seeds of the PS lines were kindly provided by Prof. Yoshihiro Matsuoka, Fukui Prefectural University. Each of these PS lines is the self-pollinated offspring of a cross between durum wheat (*T. turgidum* L. var. *durum* cv. ‘Langdon’, LDN) and one of 44 accessions of *Ae. tauschii* (L.) Coss. that originate from different geographical locations within its natural range (Matsuoka and Nasuda 2004) (Table 1). N61 is a broadly adapted Japanese cultivar that has been used as a standard variety in breeding programs in Japan.

**Table 1:** Primary synthetic lines used in this study and their parental *Ae. tauschii* accessions

No.	Synthetic line	*Ae. tauschii* parent	No.	Synthetic line	*Ae. tauschii* parent
1	Syn26	AE454	25	Syn50	AT55
2	Syn27	AE929	26	Syn51	AT76
3	Syn28	IG48042	27	Syn52	AT80
4	Syn29	IG126387	28	Syn53	AE1090
5	Syn30	IG131606	29	Syn54	IG47259
6	Syn31	KU20-8	30	Syn55	KU-20-10
7	Syn32	KU-2039	31	Syn56	KU-2076
8	Syn33	KU-2074	32	Syn57	KU-2078
9	Syn34	KU-2075	33	Syn58	KU-2079
10	Syn35	KU-2080	34	Syn59	KU-20-9
11	Syn36	KU-2088	35	Syn60	KU-2090
12	Syn37	KU-2092	36	Syn61	KU-2091
13	Syn38	KU-2096	37	Syn62	KU-2093
14	Syn39	KU-2097	38	Syn63	KU-2103
15	Syn40	KU-2098	39	Syn64	KU-2109
16	Syn41^a^	KU-2100	40	Syn65	KU-2132
17	Syn42	KU-2105	41	Syn66	KU-2136
18	Syn43^a^	KU-2106	42	Syn67	KU-2155
19	Syn44	KU-2124	43	Syn68	KU-2156
20	Syn45	KU-2126	44	Syn69	KU-2158
21	Syn46^a^	KU-2159	45	Syn70^a^	KU-2816
22	Syn47	KU-2159	46	Syn71	PI499262
23	Syn48	KU-2829A	47	Syn72	PI508262
24	Syn49	PI476874			

^a^Lines not used to produce the multiple synthetic derivatives

We crossed N61 as the male parent with each of the 44 PS lines and produced 44 F_1_ plants in 2011. One of the F_1_ plants (Syn 41) showed severe necrosis at the seedling stage and died. We crossed the remaining 43 F_1_ plants as male parents with N61 and produced 43 BC_1_F_1_ lines in 2012. We cultivated them and obtained BC_1_F_2_ seeds from individual plants in 2013. We took 10 seeds from each of 10 BC_1_F_1_ plants in the 43 lineages and mixed all the seeds to produce a bulk of 4300 seeds. We named this population MSD BC_1_F_2_. We sowed the 4300 seeds in individual plots in 2013 and obtained BC_1_F_3_ MSD seeds in 2014 from 3383 surviving plants. We randomly selected 1000 seeds and produced the BC_1_F_4_ MSD population that consisted of 983 surviving plants in 2015. In the same year, we randomly selected 400 plants and made 400 BC_1_F_5_ MSD lines as a representative population to evaluate the efficiency of our proposed platform. All this work was conducted at the Arid Land Research Center, Tottori University. In addition to the 400 BC_1_F_5_ MSD lines (hereafter the MSD population) and N61, 47 PS lines and two cultivars, Chinese Spring (CS) and LDN were used in the study. Out of the 47 PS, 43 are those used to produce the MSD population and four used as negative checks to identify the pedigree of the MSD individuals. CS was used as it represents the wheat reference genome sequence, and LDN is a tetraploid cultivar (2*n* = 4*x* = AABB) and the AB genome progenitor of the PS lines.

### Genotyping of the MSD population

Total genomic DNA was extracted using the CTAB method (Saghai-Maroof et al. 1984), and DNA samples (20 µl; 50–100 ng µl^−1^) were sent to Diversity Arrays Technology (DArT) Pty. Ltd, Australia (http://www.diversityarrays.com) for a whole-genome scan with DArTseq (DArT sequencing) markers.

### Phenotypic evaluation of the MSD population

To validate the suitability of the MSD population for wheat breeding, QTL analysis and gene identification, we scored glume coloration as a qualitative trait and heading date as a quantitative trait. Glume color was evaluated as black or not in the MSD lines, N61, LDN and the 43 PS lines at the field of the Arid Land Research Center. To evaluate heading date, the 400 MSD lines were grown during winter season in two different environments in Sudan: Dongola (19°08′N, 30°27′E, 239 masl), which is located in the Northern State and had relatively cool temperature (2721 growing degree days), and Wad Medani (14°24′N, 29°33′E, 407 masl), which is located in the Gezira State. Wad Medani was warmer than Dongola (2806 growing degree days), Seeds were sown in Dongola on 25th of November, whereas in Wad Medani on 15th of December. The experiments were arranged in an augmented randomized block design. The plot contained four 0.5 m-long rows with 0.2 m interspacing.

### Genome-wide association (GWA) analysis

A modified linear mixed model-assisted GWA analysis was conducted with the kinship matrix as a covariate in FaST-LMM (version 2.07). Kinship matrix based on the whole-genome genotypes was built using the A.mat() function of the rrBLUP (version 4.4; Endelman 2011) in R. Bonferroni-adjusted significance (adjusted *P* < 3.91 × 10^−6^) was used as the threshold to determine the significant association.

### Genome mapping and homologous gene search

To convert a wheat linkage map into a physical map, each DArT marker sequence was aligned to the wheat reference genome (version 1; IWGSC 2014) by using BLASTN. Only unique hits that perfectly matched the genome without any gaps were accepted. Wheat homologs of rice tyrosinase-related genes were estimated upon BLASTPBLASTX search with the specific criteria (E-value < 1.0 × 10^−50^ and score ≥ 200).

## Results

### General statistics of DArTseq analysis

We obtained a total of 47,994 dominant silico-DArT (SD) markers scored as presence or absence and 20,046 co-dominant SNP markers. The genetic positions of 8822 SD and 6794 SNP markers were determined on the 21 wheat chromosomes.

Among the SD markers, the frequency of genotype A (absence or presence of the SD reference sequence) was ca. 50% in most of the individuals (Fig. S1a), but was 3.8% in line MSD291. Subsequent analysis revealed that more than 35% of the MSD291 genotypes were missing (missing data), whereas other individuals had only ca. 10% of such missing genotypes (Fig. S1b). Among the SNP markers, the frequency of homozygous SNPs peaked at about 60% (Fig. S2a). The frequency of missing genotypes varied greatly (up to 59.5%), with a peak at about 7% (Fig. S2b). Missing genotype frequency in LDN was 49.8%, which was expected because LDN has no D genome. Missing genotype frequency was higher in MSD242, MSD331, MSD257 and MSD291 than in LDN. Unlike MSD291, which already presented large number of missing genotypes in SD, other three individuals had an average missing genotype ratio. The data of MSD291 was highly distorted and was discarded from further analyses.

### Genome structure of primary synthetic wheat (PS) lines

**Fig. 1:**
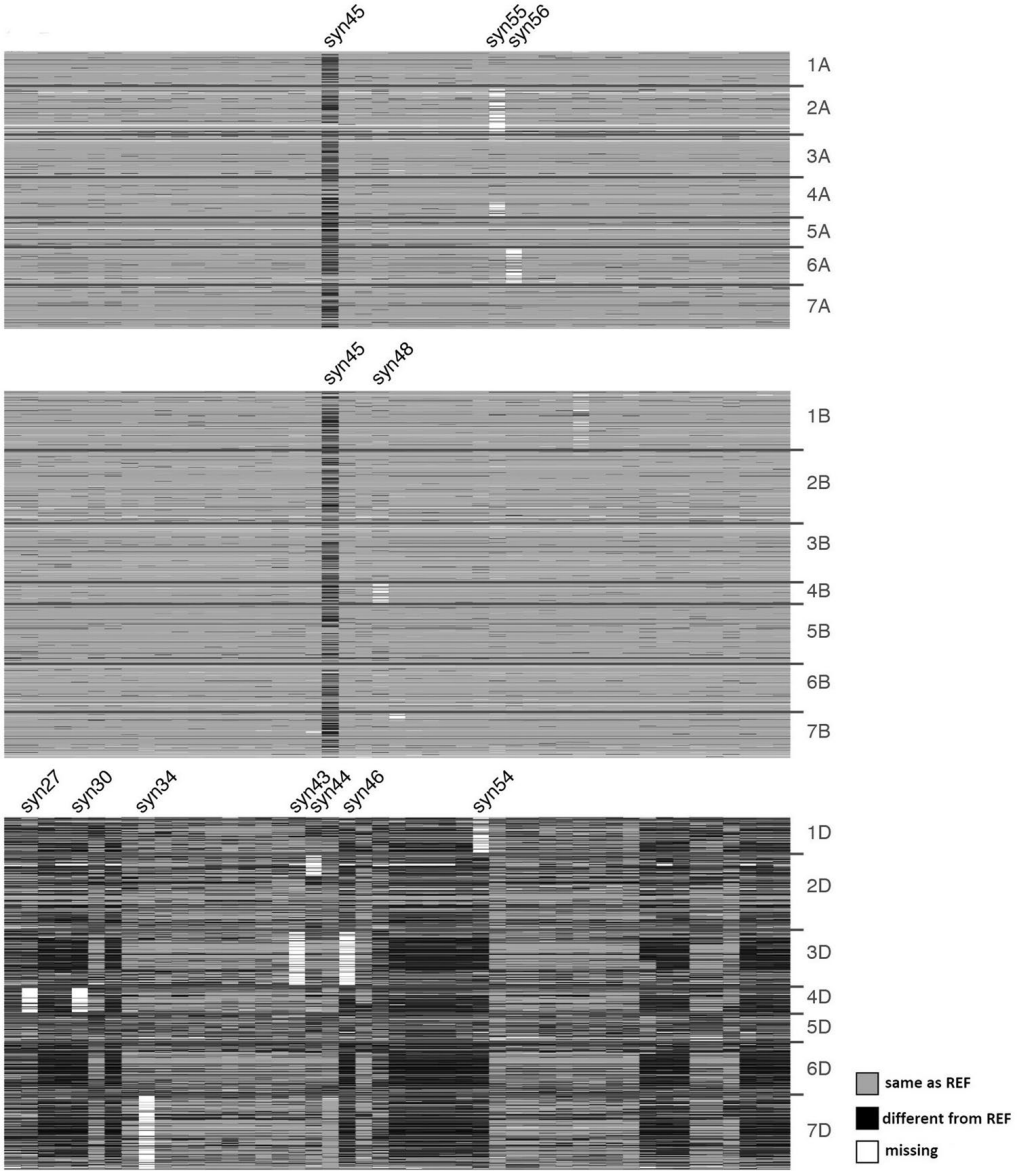
DArTseq genotyping of primary synthetic (PS) wheat lines. Graphical genotyping shows 6794 SNP markers evaluated for 47 PS lines, ‘Langdon’ as a reference for the A and B genomes and ‘Norin 61’ as a reference for the D genome. Each column indicates the genotype of each PS line. Chromosomes are separated by horizontal lines. REF: reference sequence; missing: missing genotype

To validate the accuracy of DArTseq genotyping, we graphically genotyped LDN, CS, N61 and the 47 PS lines with the 6794 SNP markers. As expected, LDN lacked the D genome and the PS lines were similar to LDN in A and B genomes, but had diverse D genomes. The band patterns of CS, N61 and LDN were distinct from each other (Fig. 1). This result indicated that DArTseq analysis was accurate.

Among the PS lines, the band patterns on A and B genomes were almost identical to each other and to that of LDN, except that the pattern of Syn45 differed from those of LDN, CS and N61 for all the three genomes (Fig. 1). This indicates that the progenitor of Syn45 was not one of these three cultivars but an unknown contaminant. Eight of the 47 PS lines had missing chromosomes, of which six were from the D genome (Fig. 1): Syn54 lacked chromosome 1D, Syn43 and Syn46 lacked chromosome 3D, Syn27 and Syn30 lacked chromosome 4D, and Syn34 lacked chromosome 7D. Using genotyping data of the D genome, we found no genetic relation among the eight lines and thus confirmed that chromosome elimination events were random (Fig. S3). Despite the presence of several nullisomics among the PS lines used to produce the MSD population, all PS lines except Syn45 had complete and pure genomes.

### The pedigree of the MSD lines

One of the purposes of DArTseq genotyping was to identify the pedigree of each of the selected MSD lines to be able to estimate the genetic drift in the population due to the selection from generation to generation. Because we had no diagnostic markers for the PS lines and the N61-originated genome fragments were distributed randomly in the MSD line genome, we developed a new method that calculated the global D genome homology between MSD individual and each PS line, after discarding markers with the same genotype as that of N61. The matched and unmatched genotypes were scored positively and negatively in the same weight. We assumed that the PS line with the highest homology score is the progenitor of the respective MSD individual. We used 2649 SD and 2403 SNP markers from the D genome; markers with a high rate of missing genotypes (less than 85% call rate) were excluded.

**Fig. 2:**
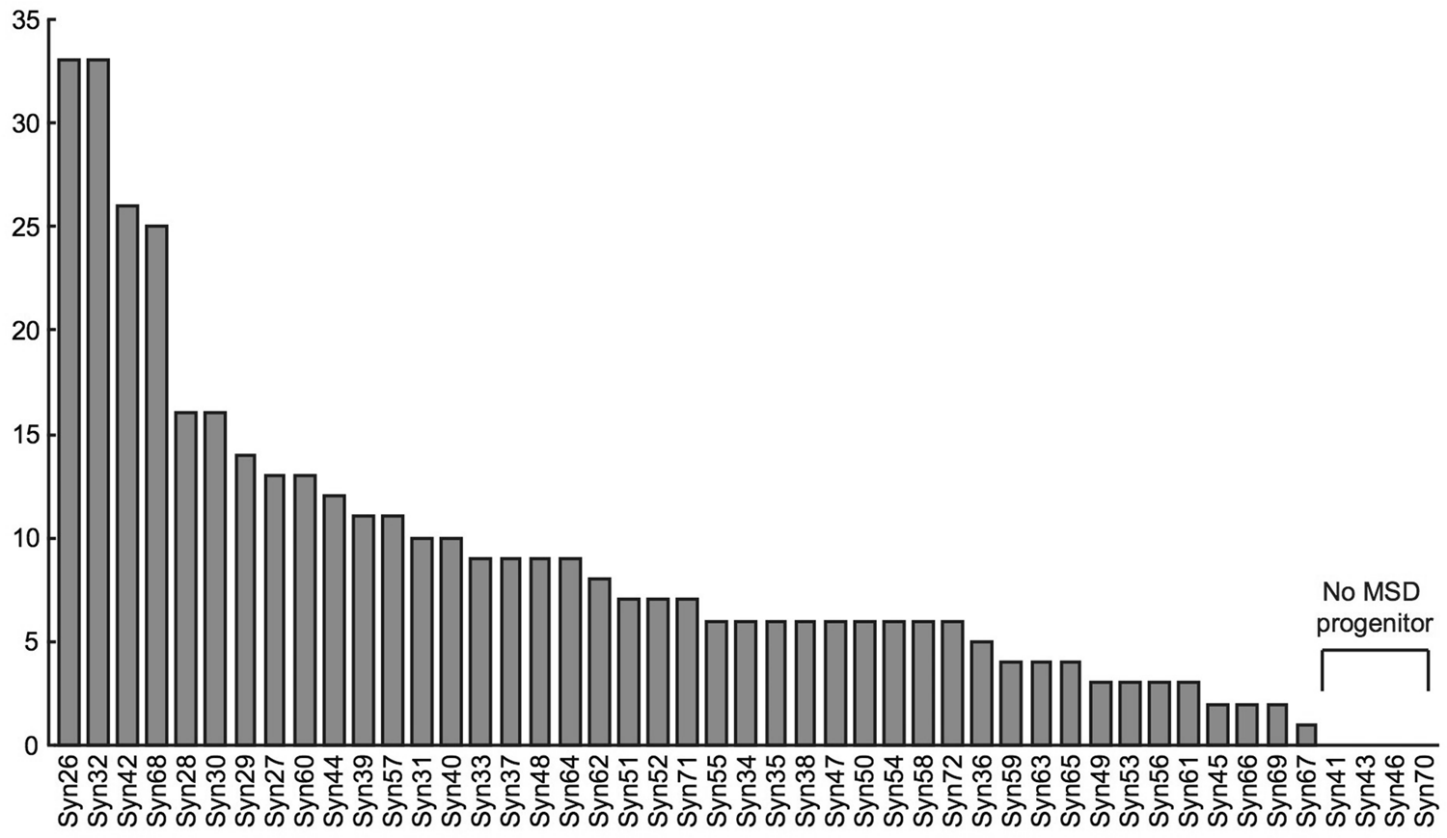
The number of progeny from each PS progenitor estimated from 399 MSD individuals. No MSD progenitor characterize the lines not used in the production of the MSD

Each of the 43 PS lines used to generate the MSD population was found to be a progenitor of at least one of the 399 MSD individuals, whereas none of the four unused PS lines (Syn41, Syn43, Syn46 and Syn70) were MSD line progenitors (Fig. 2), demonstrating that our method is quite reliable. Lines MSD108 and MSD254 were determined as progenies of the contaminated Syn45; thus, we discarded them from further analyses. Each PS progenitor produced from 1 to 33 offspring, which is wider than the expected range (5–17; single-tailed Fisher’s exact test, *P* ≥ 0.05) based on an equal contribution of each progenitor (Fig. 2). This phenomenon indicated the presence of fitness in the MSD population depending on the D genome origin. Four of the six D-chromosome nullisomic PS lines (Syn27, Syn30, Syn34 and Syn54; Fig. 1) had 41 MSD progenies out of the 397 in total. The presence of these lines (4/43 in PS vs 41/397 in MSD; *P* = 1.0, two-tailed Fisher’s exact test) and the absence of nullisomy in MSD lines indicated that nullisomy observed in the PS lines was not retained in the MSD population.

**Fig. 3:**
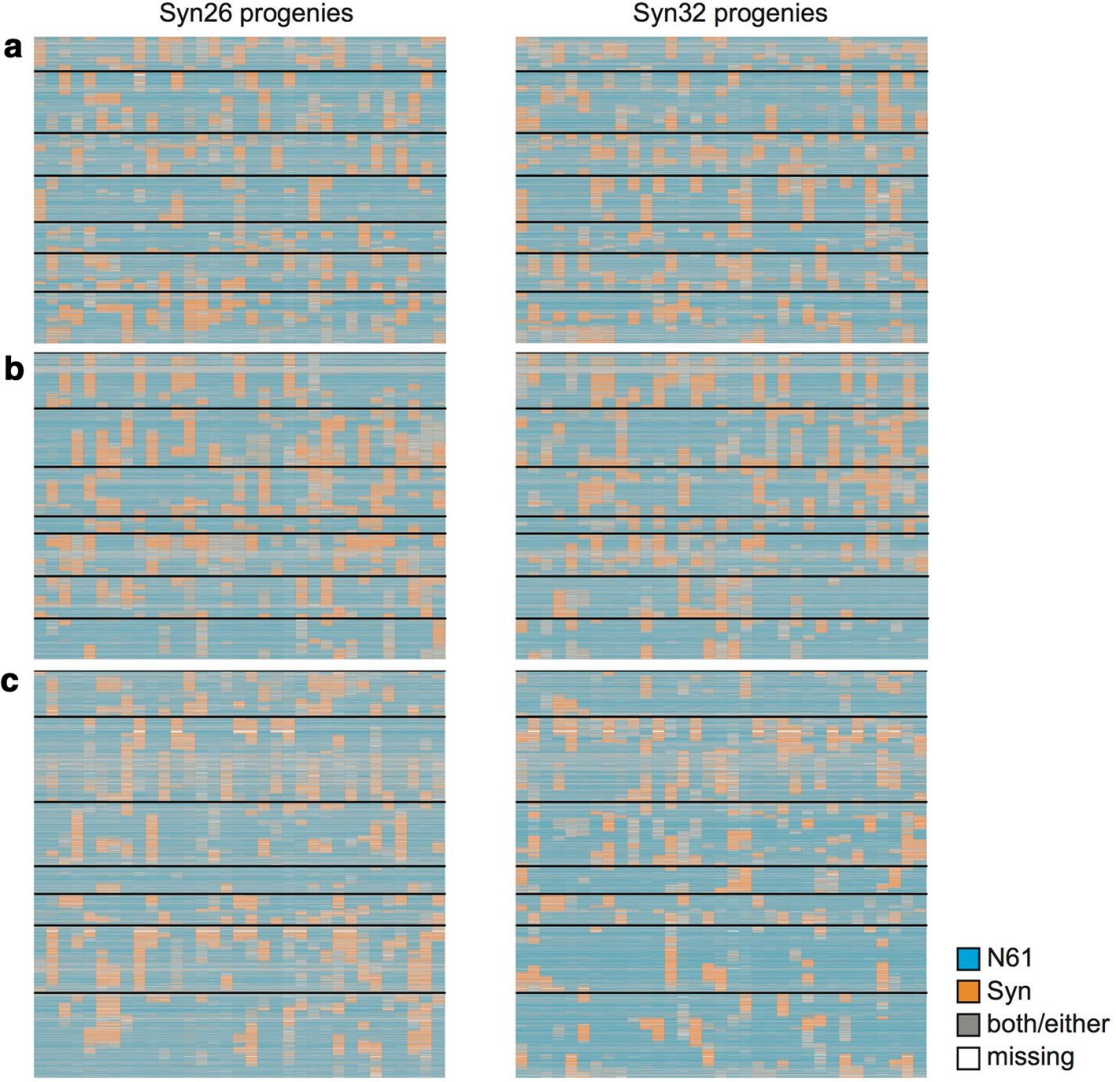
Crossing-over occurrence in the MSD population. Graphical genotyping shows DArTseq genotypes of two MSD subpopulations originating from Syn26 or Syn32, evaluated by homology to the genotype of Norin 61 (N61; blue) or the Syn line (PS; orange). Capital letters indicate wheat genomes; horizontal lines separate chromosomes

To determine whether the *Ae. tauschii* D genome affects the crossing over rate; we analyzed the crossing over status of MSD individuals. We converted the genotypes of the chromosome-assigned SD and SNP markers to the N61-like (N) or progenitor PS-like (S) form and visualized the chromosomes by using different colors for the N or S genotypes. Representative results for the MSD subpopulations that originated from the PS lines Syn26 and Syn32 are shown in Fig. 3. Regions of N61 origin prevailed in the genome, consistent with the expected 75% genome occupancy after one backcross event. Among PS genome fragments retained in the MSD genome, those in the A and B genomes originated from LDN and those in the D genome originated from *Ae. tauschii*. Random distribution and similar sizes of PS-derived fragments on D chromosomes of MSD individuals indicated that the *Ae. tauschii* genome was successfully incorporated into those of MSD lines as a result of unbiased crossing over.

In the total MSD population (excluding the three lines), the overall gene diversity (*Ht*, Nei 1987) was 0.4508, whereas the *Ht* of the D genome was 0.3633, indicating less crossing over within the D genome. Sohail et al. (2012) studied the genetic diversity and population structure of 81 *Ae. tauschii* accessions collected from different regions of its geographical distribution and classified these lines into three lineages or groups. We examined the genetic relatedness of the PS lines using the D genome markers. We found that Syn45 was placed in a separate group confirming the conclusion of the graphical genotyping that this line is a contaminant (Fig. 1). The remaining PS lines separated into three groups or lineages (Fig. S3). According to Sohail et al. (2012) Syn27, 26 and 48 are in lineage 3, Syn64–Syn66 are in lineage 2 and Syn62–Syn59 are in lineage 1. This result indicates that the PS lines represent genetic diversity from most of the *Ae. tauschii* natural habitat.

### Conversion of the DArTseq marker map positions into physical positions

**Fig. 4:**
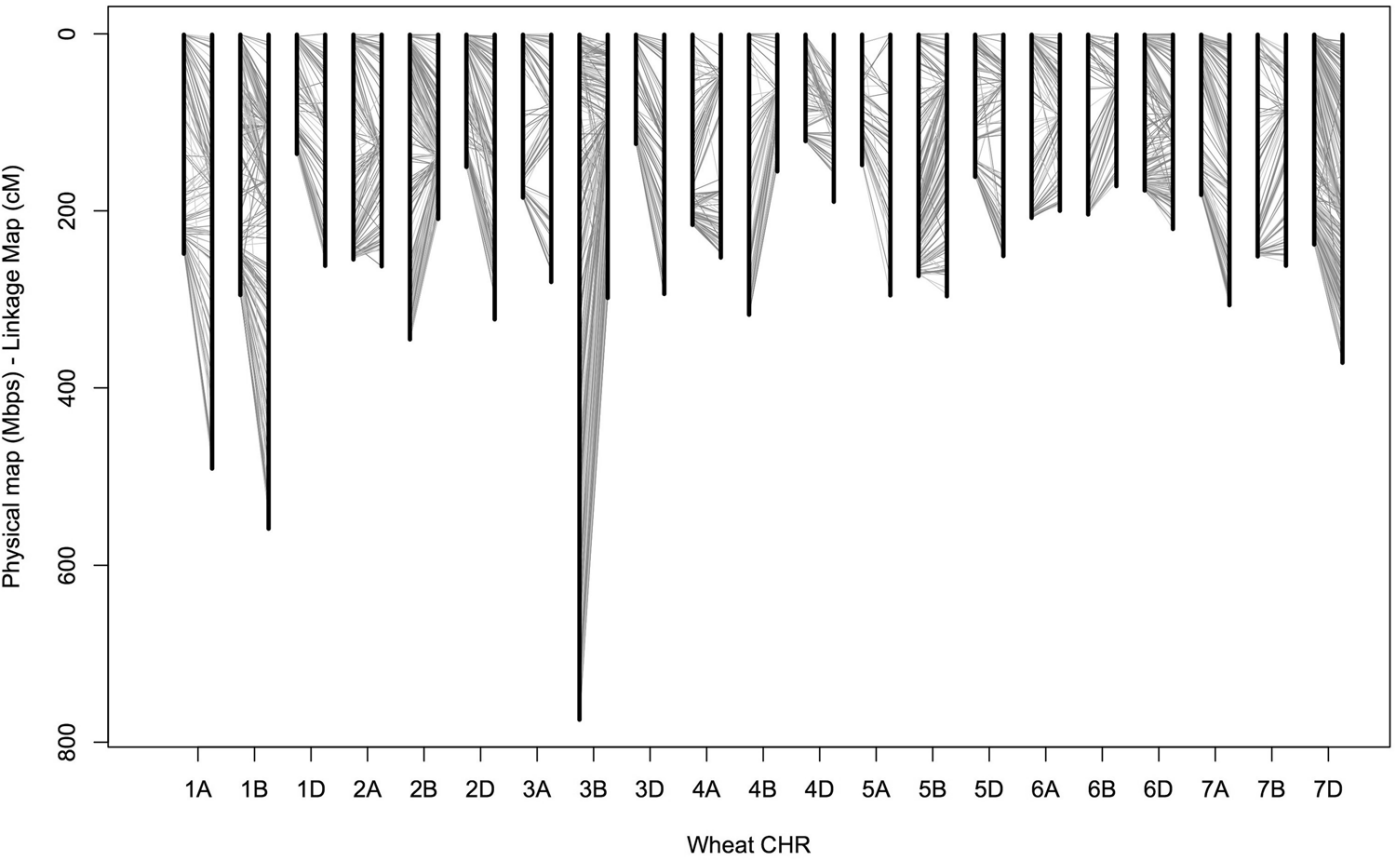
Synteny between wheat physical and linkage maps. For each chromosome (CHR), left and right vertical lines indicate physical and linkage maps, respectively. Gray lines between chromosome bars show the relative positions and order of anchored DArTseq markers

DArTseq data provided linkage distance information for a substantial fraction of markers with relatively short (28–69 nt) sequences. We converted marker linkage distances to physical positions in the wheat reference genome. A total of 14,355 marker sequences perfectly matched unique positions in the genome (Table 2). Among 15,616 chromosome-assigned SD and SNP markers, 4513 (2510 SD and 2003 SNP) markers were anchored, but only 63 of them (ca. 1.3%) were anchored between different homoeologous chromosomes. The remaining chromosome-matched markers were evenly distributed on the chromosomes, and the order of markers was generally similar in both linkage and physical maps (Fig. 4).

**Table 2:** Numbers of DArTseq markers on maps described in this study

Chr.	Linkage map		Physical map^a^		
	Number of markers	Avg. gap (cM)	Number of markers	Avg. gap (Mb)	Anchored markers
1A	511	0.96	527	0.47	147
2A	913	0.29	800	0.32	250
3A	632	0.43	311	0.60	121
4A	686	0.37	620	0.35	183
5A	461	0.64	221	0.67	75
6A	570	0.34	611	0.34	191
7A	764	0.40	533	0.34	211
1B	1102	0.50	702	0.42	275
2B	1149	0.18	1033	0.33	336
3B	973	0.31	1318	0.59	497
4B	337	0.45	440	0.72	126
5B	833	0.35	908	0.30	251
6B	831	0.21	352	0.58	125
7B	802	0.33	365	0.69	134
1D	573	0.46	641	0.21	168
2D	1057	0.29	969	0.16	198
3D	796	0.37	359	0.35	138
4D	345	0.55	487	0.25	138
5D	390	0.64	980	0.16	179
6D	837	0.26	920	0.19	316
7D	1054	0.35	1258	0.19	454
Total	15,616		14,355		4513

^a^Information from wheat reference genome version 1 (IWGSC 2014)

### GWA analysis

To evaluate the versatility of the MSD population, elucidate D genome-derived agronomic traits, and verify the suitability of the population for QTL identification, we conducted GWA analysis for glume coloration as a qualitative trait and heading date as a quantitative trait.

**Fig. 5:**
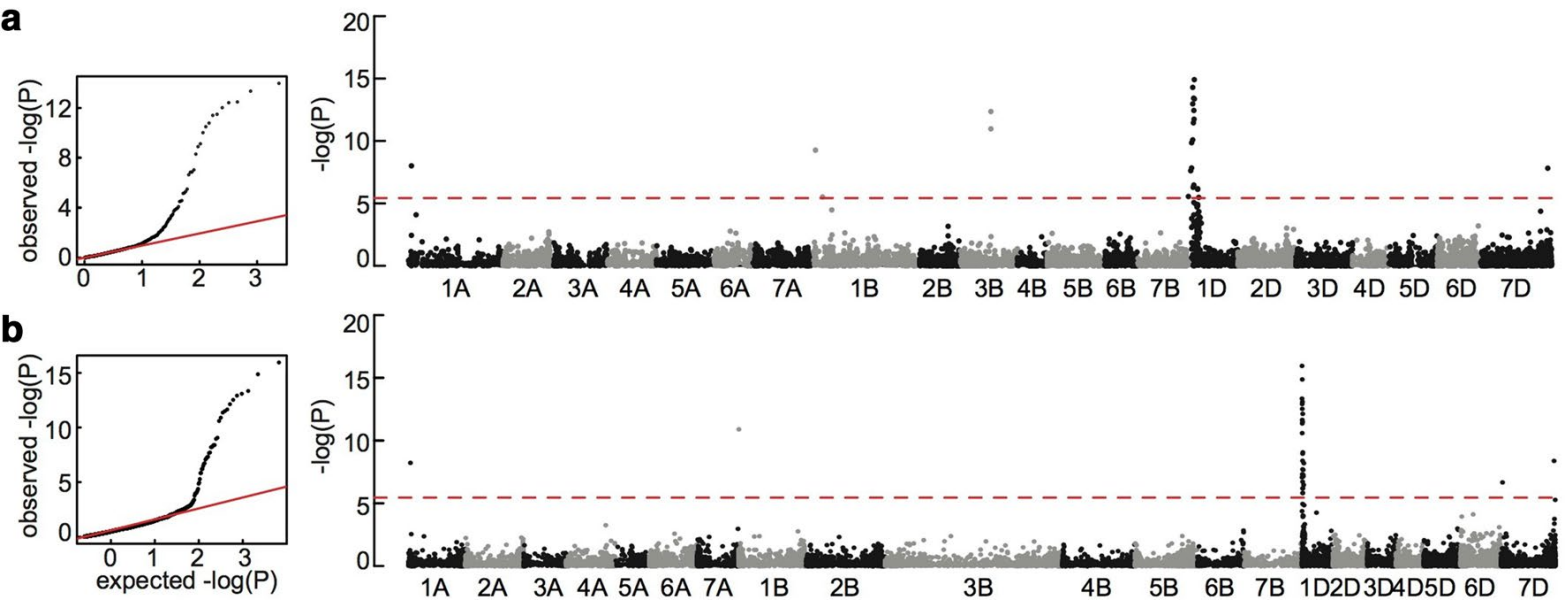
Genome-wide association (GWA) analysis of the black glume trait in the MSD population. Results of GWA analyses based on **a** linkage map and **b** physical map are presented. Left panels, quantile–quantile plots; right panels, Manhattan plots. Dashed horizontal lines in Manhattan plots correspond to the Bonferroni-adjusted significance threshold (3.91 × 10^−6^)

Glume coloration is one of the well-studied traits in wheat and other Triticeae crops. Almost all modern wheat cultivars, including LDN and N61, have colorless glume, whereas a substantial fraction of the MSD and PS lines had black glume, indicating that this trait is controlled by allele(s) from the D genome in an epistatic manner. Among the 397 MSD individuals evaluated, 336 had no spike pigmentation (similar to LDN and N61), whereas 61 (15% of the MSD population) had black spikes. Ancestry estimation indicated that 30 PS lines contributed to the black glume trait; the 61 MSD lines correspond to almost one-quarter of the 292 progenies of these PS lines (*P *= 0.61; single-tailed Fisher’s exact test). GWA analysis using the linear mixed model with both the linkage and physical maps showed a single prominent peak at 22.564 cM on the short arm of chromosome 1D (Fig. 5a), corresponding to a sharp association peak at 2.07 Mb (range, 0.3–2.28 Mb) of the wheat reference chromosome 1D (Fig. 5b); this region harbored 64 protein-coding genes (Table S1). The black glume color suggests that the pigment is melanin. Melanin biosynthesis in plants is largely regarded to tyrosinase activity (Singh et al. 2013). A model monocot plant rice contains six tyrosinase-related genes, and we identified five homologs from the current wheat genome (Table S2). However, among the 64 genes of which several encoded putative enzymes, none of these involved in melanin biosynthesis (Table S1).

**Fig. 6:**
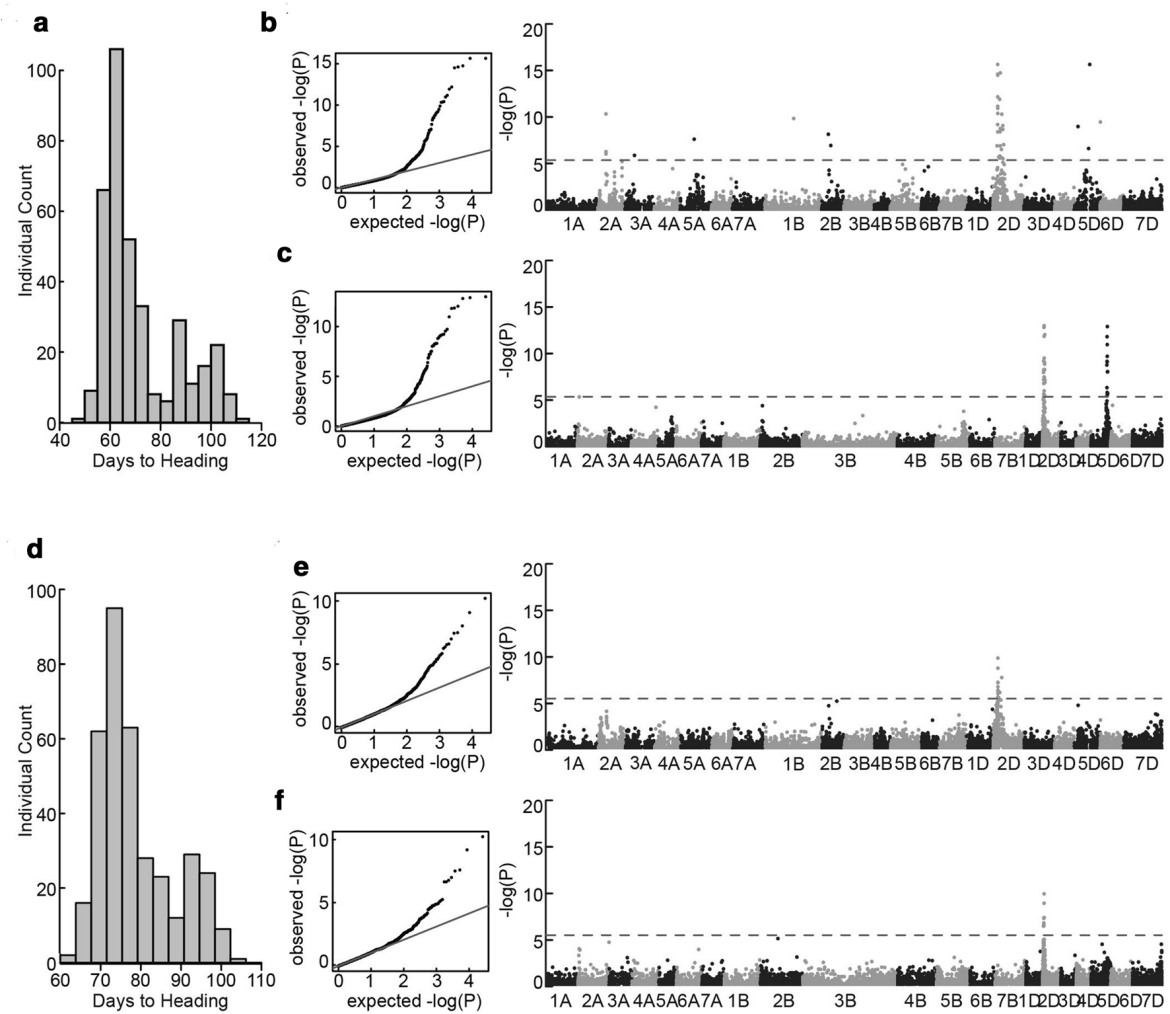
Genome-wide association (GWA) analysis of days to heading in the MSD population. **a**, **d** Distribution of days to heading in the MSD population at **a** Wad Medani **d** Dongola. **b**, **c**, **e**, **f** show the results of GWA analyses based on **b**, **e** linkage map or **c**, **f** physical map. Left, quantile–quantile plots; right, Manhattan plots. Dashed horizontal lines in Manhattan plots correspond to the Bonferroni-adjusted significance threshold (3.91 × 10^−6^)

Evaluation of heading date at Dongola showed two major peaks: the larger one at ~ 70 days (early flowering individuals) and the smaller one at ~ 95 days (late flowering individuals). The ratio of the early to the late genotypes was consistent with 3:1 (*P *= 1.0, two-tailed Fisher’s exact test), indicating that a single gene controls DH in the MSD population (Fig. 6d). At Wad Medani, three peaks (~ 60, ~ 85 and ~ 100 days) were observed (Fig. 6a) indicating that more than one gene controls the heading time.

At Dongola, GWA analysis based on either genetic or physical map revealed a single significant peak on the short arm of chromosome 2D (Fig. 6e, f). The peak was located between 47.521 and 84.625 cM, which corresponds to 11.98–13.26 Mb; this region included 55 protein-coding genes (Table S3). At Wad Medani genetic map-based analysis detected five significant peaks on the short arms of chromosomes 2A, 2B and 2D, and on the long arms of chromosomes 5A and 5D (Fig. 6b), whereas physical map-based analysis detected only two significant peaks on the short arm of chromosome 2D and the long arm of chromosome 5D (Fig. 6c).

In the two environments, a highly significant peak was detected on the short arm of chromosome 2D. The position of this peak matched that of *Ppd*-*1D*, a pentatricopeptide repeat (PPR) protein-coding gene, which strongly affects wheat response to photoperiod (Langer et al. 2014; Guedira et al. 2016). However, we did not find a PPR gene sequence within the peak range (Table S3). Our search with the previously reported *Ppd*-*1D* sequence (Guo et al. 2009) in the wheat reference genome used in this study detected no significant hits.

## Discussion

This study demonstrates the potential of a novel genetic platform, the MSD population, to be used for thorough investigation of novel traits and QTLs from the wild wheat relative *Ae. tauschii*. We have described the development of the MSD population and revealed its value for the wheat research community.

The absence of correlation between *Ae. tauschii* traits and those of the corresponding hexaploid synthetics has been reported (Sohail et al. 2011; Fujiwara et al. 2010; Kurahashi et al. 2009). Therefore, we need to identify the genes of *Ae. tauschii* that confer desirable traits in synthetics. Synthetics usually show phenotypes different from those of cultivated bread wheat, which limits accurate evaluation of yield potential; moreover, sometimes the target traits do not express in elite wheat backgrounds (Ogbonnaya et al. 2013) which necessitate development of populations containing large diversity of *Ae. tauschii* in elite wheat backgrounds. We developed an MSD population of 4300 lines and genotyped a small part of this population (400 lines) to reveal its value. Using the DArTseq platform, we identified the pedigree of each of the 400 lines. This information will facilitate tracking the desired traits from the original PS line and using it in further crosses. The markers allowed us to ensure the purity of the material and to exclude problematic lines.

The original 4300 MSD population consisted of 100 offspring of each of the 43 distinct *Ae. tauschii* accessions. This population enables us to rapidly select desired plants or phenotypes from the diversity of 43 *Ae. tauschii* accessions. In the MSD population, the A and B genomes came from LND (25%) and N61 (75%), which allows more systematic analysis of the genetic diversity of *Ae. tauschii* than in the traditional system, which usually involves complex crosses and pedigrees. Because the A and B genomes have originated from either LDN or N61, the QTLs and genes in these genomes can be easily identified as in ordinary bi-parental mapping populations. Our MSD population can facilitate analysis of variation of the D genome more systematically as the A and B genomes are identical among the MSD individuals, and thus mimics diploid wheat. However, we need to consider the interaction between genes on D genome and A or B genome. Double backcrossing to N61 has narrowed the genetic and morphological distance between synthetic wheat and modern cultivars (i.e., N61), with a wider diversity in the D genome than in the A and B genomes. This characteristic of the MSD will facilitate the use of genomic information of the reference cultivar CS.

Multi-parent advanced intercross (MAGIC) populations with four or eight founders (Huang et al. 2012; Mackay et al. 2014) and nested association mapping (NAM) populations (Bajgain et al. 2016) have been developed to solve the problem of the narrow genetic base of bread wheat and to increase the efficiency of the detection of desired genes or QTLs. In MAGIC populations, multiple founders contribute more allelic diversity than that captured in bi-parental populations, and multiple cycles of intercrossing give greater opportunities for recombination and, hence, greater precision in QTL location (Mackay et al. 2014). NAM populations facilitate both linkage analysis and association mapping. In both population types, breeders tend to use alleles currently available within the elite wheat germplasm and to use wild relative’s alleles in a narrow range, whereas the MSD population was developed mainly to explore and harness the diversity of wild wheat progenitors by using a bigger set of wild accessions and hence is more genetically diverse.

The gene diversity values of the MSD population are relatively high compared to those reported by Mackay et al. (2014) for an eight parent MAGIC population and founders. On the other hand, the analysis of the genetic relatedness among the PS lines (Fig. S3) was consistent with the results of Sohail et al. (2012) and revealed that the MSD population represents genetic diversity from most of the *Ae. tauschii* natural habitat. Currently, we are attempting to increase the outcrossing rate among the MSD individuals by chemical application or introduction of genetic male sterility to increase the recombination to maximize the genetic diversity and aid the precise mapping of genes and QTLs in the MSD population.

Although we mixed the same number of BC_1_F_2_ seeds from each pedigree, the numbers of the descendants found in the MSD population were greatly different depending on the family (Fig. 2). This may attribute to natural selection in the process of repeated self-pollination procedure in the field to make the MSD population. The difference in adaptability may be growth competitiveness among plants and seed productivity, or weakness seen frequently in the hybrids. However, we could not find any relationship between the fitness to produce more descendants and the phylogeny of *Ae. tauschii* (Fig. S3).

The genome of each MSD individual contains one-fourth of the *Ae. tauschii* wild genome, whose fragments are distributed randomly, and three-quarters of N61 genome (Fig. 3). Theoretically, we estimated that, to cover the whole genetic information of *Ae. tauschii*, at least 9 BC_1_F_1_ individuals from a single PS line are required. From each BC_1_F_1_ plant, 10 BC_1_F_2_ plants were selected, and 100 BC_1_F_2_ plants were produced from each cross with the assumption that they cover all the genome of the *Ae.tauschii* used to produce the PS line. With this assumption, to screen all the 43 *Ae. tauschii* (43 PS) genome information, we propose that a 4000 MSD population would be needed.

We evaluated glume coloration as a qualitative trait and DH as a quantitative trait to validate the suitability of the MSD population for QTL identification and isolation of *Ae. tauschii* genes. One QTL for glume color was identified on the short arm of chromosome 1D (Fig. 5). In wheat glume coloration is controlled by alleles at the short arms of chromosomes 1AS (*Rg*-*A1*), 1BS (*Rg*-*B1*) and 1DS (*Rg*-*D1*) (Khlestkina et al. 2006, 2009). Multiple alleles were reported for *Rg*-*A1* and *Rg*-*D1* beside the colorless wild-type allele. Alleles *Rg*-*A1b* and *Rg*-*A1c* from *T. aestivum* control red and black glume coloration, respectively, whereas *Rg*-*D1b* (*Ae. tauschii*) and *Rg*-*D1c* (*T. aestivum*) control red and smokey-gray colors, respectively (Efremova et al. 1998; Arbuzova et al. 1998; Jones et al. 1990; Börner et al. 2002; Pshenichnikova et al. 2005). LDN and N61 that used to produce the PS and the MSD population had no spike pigmentation, whereas some of PS and *Ae. tauschii* showed black spikes indicating that *Ae. tauschii* is the source of the black glume observed in the MSD individuals. On the other hand, the identified alleles at the *Rg*-*D1* locus control red and smokey-gray pigmentation which are different from the black pigmentation observed in our study. Taking these together it is possible for us to speculate that the black pigmentation observed in our study is controlled by a new allele at the *Rg*-*D1* locus, but this speculation need to be confirmed in future studies. Thus, using the MSD population, we were able to correctly map the glume coloration QTL. However, although we detected 64 protein-coding genes (Table S1), we could not identify the causal gene.

At Dongola, we identified one QTL for heading date on the short arm of chromosome 2D; the position of this QTL corresponded to that of the well-known photoperiod allele *Ppd*-*1D* (Langer et al. 2014; Guedira et al. 2016). At Wad Medani, we identified five QTLs (Fig. 6). Three QTLs corresponded to the well-known photoperiod alleles *Ppd*-*1A*, *Ppd*-*1B* and *Ppd*-*1D* (Langer et al. 2014; Guedira et al. 2016), and the remaining two the vernalization loci *Vrn*-*A1* and *Vrn*-*D1* (Kippes et al. 2014). The differences in temperature between the two environments might explain the difference in the QTLs detected in each case. At Dongola, where the temperature was cool, the photoperiod response controlled heading time and no vernalization loci were involved. At Wad Medani, which was warmer than Dongola, heading was controlled by both the photoperiod and vernalization.

Using both genetic and physical maps, we mapped one highly significant QTL at the locus of the photoperiod allele *Ppd*-*1D* on the short arm of chromosome 2D (Langer et al. 2014; Guedira et al. 2016) in both environments (Fig. 6). Our failure to identify the causal gene among the 55 genes identified within the QTL range or by using the reported sequence of the *Ppd*-*1D* gene suggests that the annotation of this gene is missing from the present reference wheat genome because it is an incomplete draft sequence (IWGSC 2014). Because 75% of the A and B genomes of the MSD lines originated from N61, the N61 genome could be the genetic source of the early heading phenotype, and the late phenotype could be due to a wild allele of *Ppd*-*1D* originated from the *Ae. tauschii* genome. This assumption is plausible because the heading time was observed under short-day conditions and *Ppd*-*1D* alleles are major regulators of wheat photoperiod response.

Nguyen et al. (2013) reported three QTLs for heading time on chromosomes 1D, 6D and 7D in F_2_ populations involving four of the PS used to develop the MSD population. However, none of these QTLs was identified in this study. This could be due to several reasons; (1) the difference of the environments used in both studies, (2) the strong effect of the *Ppd*-*1D* of N61 (Nguyen et al. 2015), and/or (3) the fact that each of the PS lines was represented with small number of genotypes in the total 400 MSD lines studied.

Our results revealed that the MSD population allows efficient identification of new QTLs or genes from *Ae. tauschii* that could be used to improve both quantitative and qualitative traits in wheat. Previously, from the MSD population, we identified several highly heat-tolerant lines and several other lines that contained alleles able to enhance yield potential (Elbashir et al. 2017a, b). Currently, these 400 lines are being evaluated for salinity tolerance and phosphorus deficiency tolerance.

## Conclusions

The use of the MSD platform is an effective strategy to harness the huge and valuable diversity present in the *Ae. tauschii* genome. Using the MSD population, which represents the diversity of 43 *Ae. tauschii* accessions, QTLs, genes and desired phenotypes could be identified and selected for use in wheat breeding. This population is available upon request from the Laboratory of Arid Land Plant Resources of the Arid Land Research Center of Tottori University, Japan.

### Author contribution statement

YSAG, JSK and HT conceived and designed the experiment. AAEA performed the phenotypic analysis and DNA extraction. JSK performed the genotypic analysis and contributed to the manuscript drafting. YSAG interpreted the results and drafted the manuscript. HT supervised the study. All authors read and approved the final manuscript.

## Electronic supplementary material

Below is the link to the electronic supplementary material.

Fig. S1 Genotype frequency of silico-DArT (SD) markers in individuals from the MSD population (a) and the frequency of the missing genotypes (b)

Fig. S2 Genotype frequency of SNP markers in individuals from the MSD population (a) and the missing genotype frequency (b). The bin size was a frequency of 0.01

Fig. S3 Dendrogram based on the genotypic data of the D genome showing genetic relatedness among primary synthetic lines

Supplementary material 4 (XLSX 14 kb)

Supplementary material 5 (XLSX 9 kb)

Supplementary material 6 (XLSX 12 kb)
