# Supplementary figures and images for: A population of wheat multiple synthetic derivatives: an effective platform to explore, harness and utilize genetic diversity of Aegilops tauschii for wheat improvement
Source: Theor Appl Genet. 2018 Apr 28;131(8):1615–26. doi: 10.1007/s00122-018-3102-x (PMC6061144; doi:10.1007/s00122-018-3102-x)

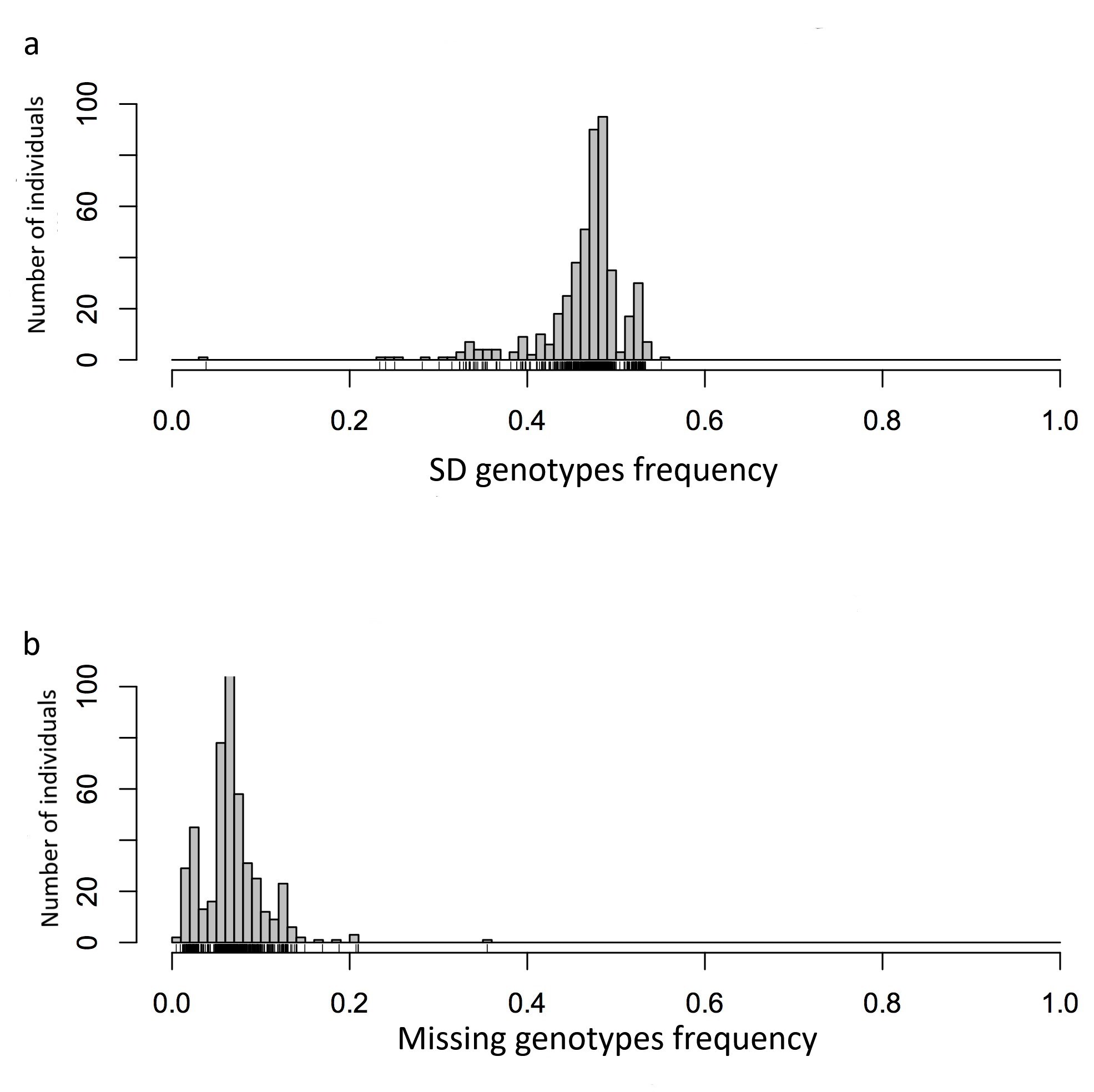

Supplement: Supplementary file 1 — Fig. S1 Genotype frequency of silico-DArT (SD) markers in individuals from the MSD population (a) and the frequency of the missing genotypes (b) [file 122_2018_3102_MOESM1_ESM.jpg]

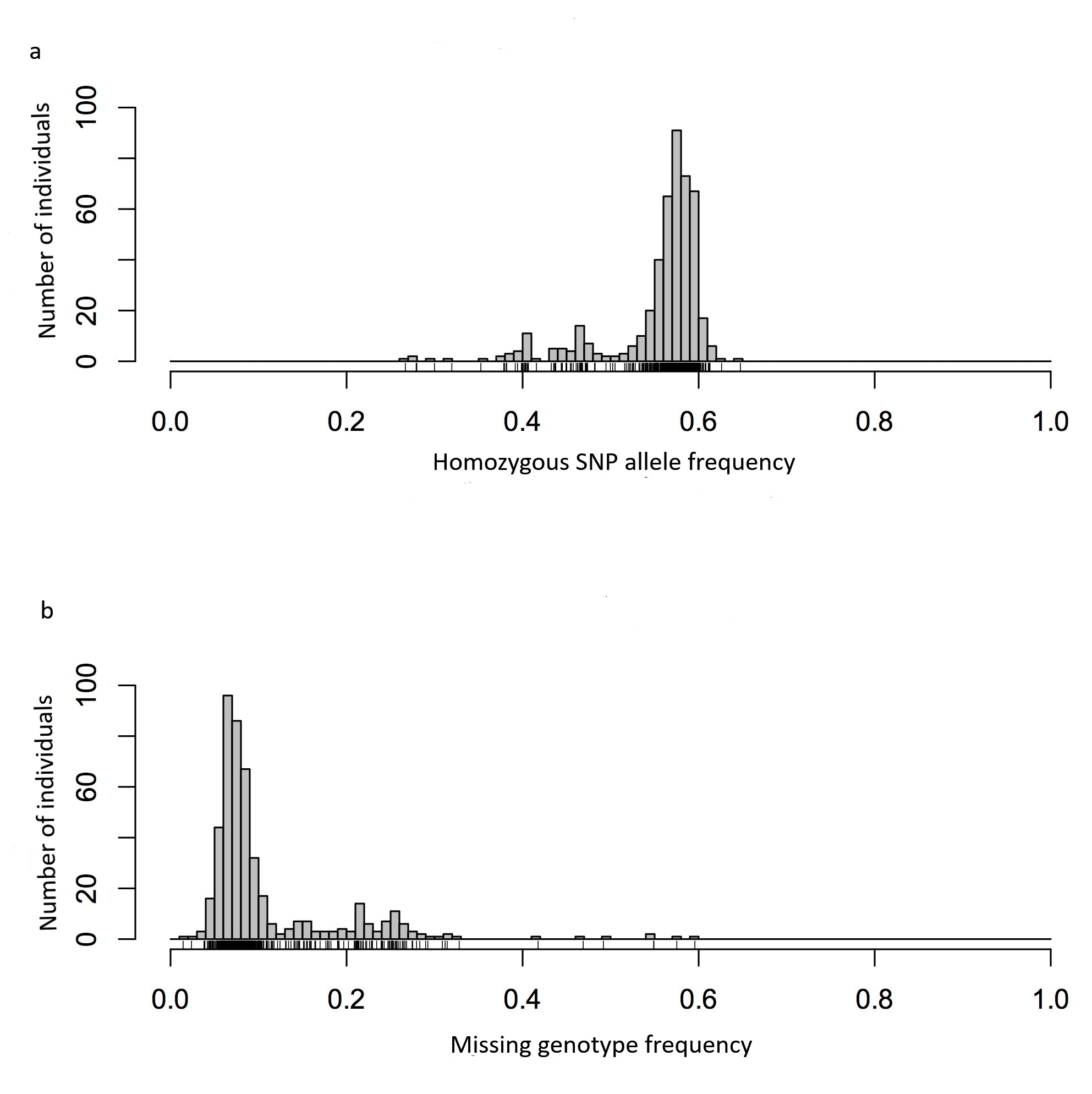

Supplement: Supplementary file 2 — Fig. S2 Genotype frequency of SNP markers in individuals from the MSD population (a) and the missing genotype frequency (b). The bin size was a frequency of 0.01 [file 122_2018_3102_MOESM2_ESM.jpg]

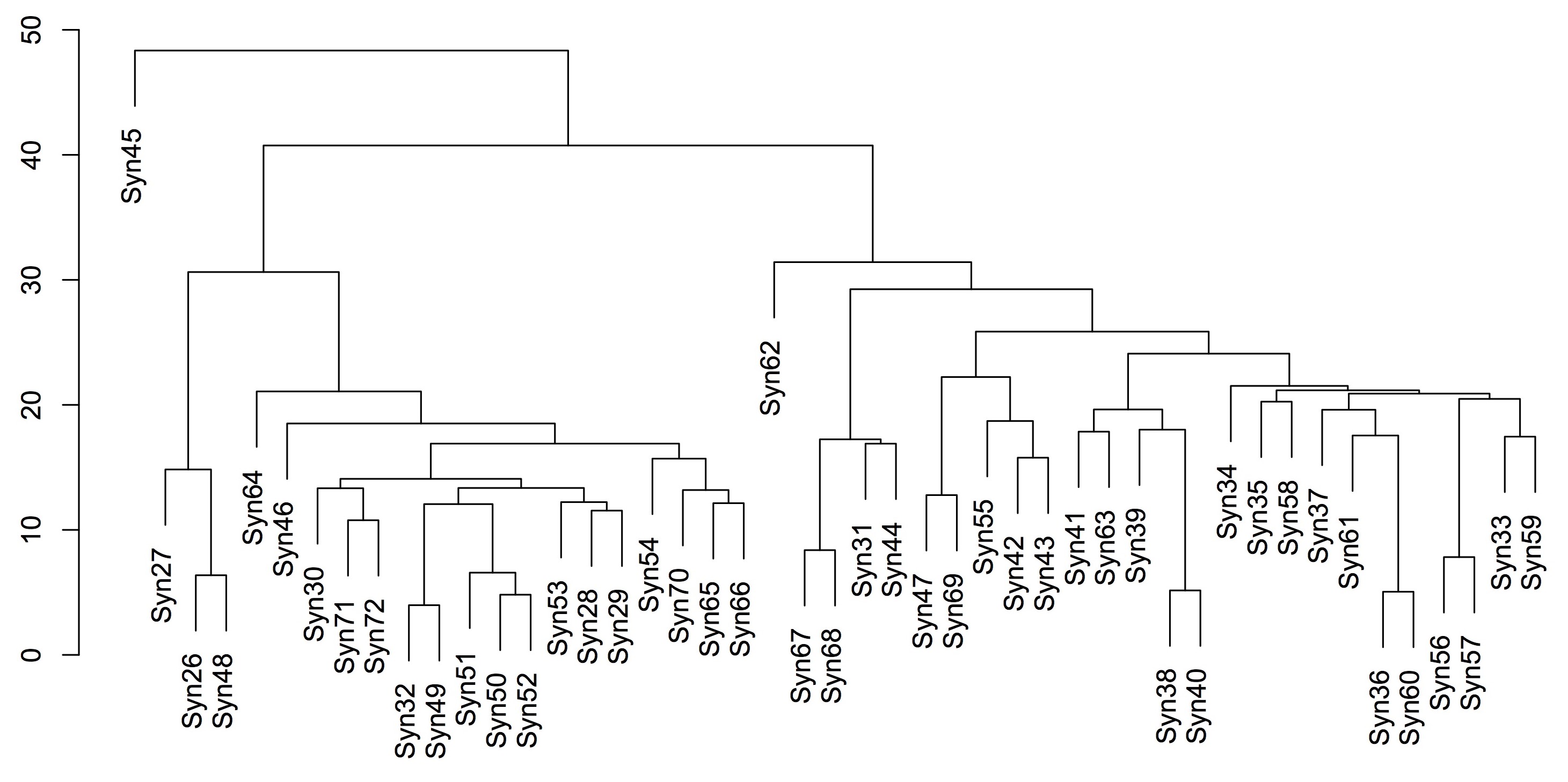

Supplement: Supplementary file 3 — Fig. S3 Dendrogram based on the genotypic data of the D genome showing genetic relatedness among primary synthetic lines [file 122_2018_3102_MOESM3_ESM.jpg]
